# Activity pacing for osteoarthritis symptom management: study design and methodology of a randomized trial testing a tailored clinical approach using accelerometers for veterans and non-veterans

**DOI:** 10.1186/1471-2474-12-177

**Published:** 2011-08-02

**Authors:** Susan L Murphy, Angela K Lyden, Maria Clary, Michael E Geisser, Raymond L Yung, Daniel J Clauw, David A Williams

**Affiliations:** 1 Department of Physical Medicine and Rehabilitation, University of Michigan, Ann Arbor, MI, USA; 2 VA Ann Arbor Healthcare System, GRECC, Ann Arbor, MI, USA; 3 Department of Internal Medicine, Division of Geriatric Medicine, University of Michigan, Ann Arbor, MI, USA; 4 Department of Anesthesiology, University of Michigan, Ann Arbor, MI, USA

## Abstract

**Background:**

Osteoarthritis (OA) is a prevalent chronic disease and a leading cause of disability in adults. For people with knee and hip OA, symptoms (e.g., pain and fatigue) can interfere with mobility and physical activity. Whereas symptom management is a cornerstone of treatment for knee and hip OA, limited evidence exists for behavioral interventions delivered by rehabilitation professionals within the context of clinical care that address how symptoms affect participation in daily activities. Activity pacing, a strategy in which people learn to preplan rest breaks to avoid symptom exacerbations, has been effective as part of multi-component interventions, but hasn't been tested as a stand-alone intervention in OA or as a tailored treatment using accelerometers. In a pilot study, we found that participants who underwent a tailored activity pacing intervention had reduced fatigue interference with daily activities. We are now conducting a full-scale trial.

**Methods/Design:**

This paper provides a description of our methods and rationale for a trial that evaluates a tailored activity pacing intervention led by occupational therapists for adults with knee and hip OA. The intervention uses a wrist accelerometer worn during the baseline home monitoring period to glean recent symptom and physical activity patterns and to tailor activity pacing instruction based on how symptoms relate to physical activity. At 10 weeks and 6 months post baseline, we will examine the effectiveness of a tailored activity pacing intervention on fatigue, pain, and physical function compared to general activity pacing and usual care groups. We will also evaluate the effect of tailored activity pacing on physical activity (PA).

**Discussion:**

Managing OA symptoms during daily life activity performance can be challenging to people with knee and hip OA, yet few clinical interventions address this issue. The activity pacing intervention tested in this trial is designed to help people modulate their activity levels and reduce symptom flares caused by too much or too little activity. As a result of this trial, we will be able to determine if activity pacing is more effective than usual care, and among the intervention groups, if an individually tailored approach improves fatigue and pain more than a general activity pacing approach.

**Trial Registration:**

ClinicalTrials.gov: NCT01192516

## Background

Osteoarthritis (OA) is a prevalent and chronic disease. It affects over 27 million adults in the U.S. and is expected to increase as the population ages [1]. OA is more prevalent among females, however, male veterans are also significantly affected, with 1/3 of males of all ages diagnosed and nearly half of those who use the Veteran's Affairs (VA) healthcare system [2]. OA causes substantial physical and psychosocial disability. By 2020, it is expected that 12 million people in the U.S. will report activity limitations due to arthritis [3].

A primary reason people with OA seek treatment is for pain relief. Whereas people with OA often receive pharmacological treatment for pain reduction, non-pharmacological treatment for symptoms is considered the foundation of OA management [4,5]. Rehabilitation professionals, such as occupational therapists (OTs), can tailor non-pharmacological symptom management interventions so as to promote adherence and behavior change. To date, however OTs have had only limited involvement in tailoring such interventions for people with OA. More research is needed on the effectiveness of these tailored symptom management interventions when delivered by rehabilitation professionals within the context of clinical care. Well-established community-based programs on self-management strategies (such as the Arthritis Self-Management Program) have been shown to have small but positive effects on pain and arthritis self-efficacy [6-8]. Although community-based programs are an important tool for OA management, such programs do not optimize the use of rehabilitation professionals in the delivery or tailoring of these programs.

### Behavior Change and Tailoring Interventions

A tailored approach to interventions is being increasingly recommended in order to make the content personally relevant and to promote long-term behavior change. Several interventions are individually tailored on key variables identified in theoretical health behavior models, such as motivational readiness, decisional balance, or self-efficacy [9-11]. Interventions also have been tailored on personal characteristics (e.g., gender [12], depression [13], pain level [14]). There is growing evidence that tailoring enhances the effectiveness of clinical interventions designed to alter health risk behaviors such as smoking [15,16] and low physical activity [17,18].

For arthritis management interventions, Social Cognitive Theory has often been used as the theoretical foundation of intervention development [19,20]. It posits that people learn through an interaction of social and environmental influences that contribute to their own motivations and behaviors [21]. To adopt a new health behavior, the central determinants include 1) *knowledge *of the risks and benefits, 2) *outcome expectations *about costs and benefits for engaging in health behaviors, 3) the health *goals *people set for themselves and plans for goal achievement, 4) the *perceived facilitators and barriers *(such as external social or work demands), and 5) *self-efficacy*, the belief in the ability to perform under a certain set of conditions [21,22]. Self-efficacy is often a central focus in self-management interventions because it influences behavior both directly and indirectly through the other determinants above [22]. Numerous studies have found that improvements in pain coping skills are related to improvements in arthritis self-efficacy [19,23,24].

In this study, the intervention is designed to assist people in enhancing their arthritis self-efficacy by individually tailoring the use of activity pacing strategies to the context of where and when the strategies may be most useful. According to Social Cognitive Theory, self-efficacy is enhanced by building competency through practice (and by the rewards associated with success) in specific situations or conditions [22]. By tailoring activity pacing instruction to individuals' real-life routines and activities, there is a greater chance for learning to occur and for self-efficacy to be enhanced given the greater proximity of the pacing schedule to real-life and personally relevant activities.

### Pain, Fatigue, and Their Relationship to Physical Activity

Both pain and fatigue symptoms are linked to various functional problems in OA. Pain in OA affects the ability to engage in activities of daily living, work, and other meaningful activities and is associated with a reduced quality of life [25-27]. Fatigue is not as well-studied in OA, however, it is one of the most frequently reported OA symptoms [28,29]. Attention to the clinical importance of fatigue is growing. In large studies, substantial fatigue was reported by 41% of patients with OA [28], and problematic fatigue or tiredness was reported in moderate levels [30]. Fatigue in OA is one of the strongest predictors of functional disability [28,31] and work dysfunction [28]. Fatigue has also been associated with pain, sleep problems, functional disability, and depression [28,30-32]. Qualitative studies have further characterized the fatigue experience in OA as debilitating, causing activity restriction, and greatly impacting life [29,33].

A better understanding of how symptoms affect activity on a more micro level, such as over a day, week, or month, can help refine and tailor OA interventions. For example, tailored interventions may target *timing *of behaviors such as medication usage or variability in symptoms by time [34]. Although OA symptoms are believed to be less variable than other rheumatic conditions such as rheumatoid arthritis (RA), an emerging body of research has shown that there is important variability in OA pain [35-38] across days or even within a day. These pain fluctuations have been associated with limited daily activities, days of missed work, sleep interference, and mood [36], and increased pain variability may be associated with personal factors [e.g., body mass index (BMI)] [35].

Fewer studies have examined fatigue variability in OA. Given the strong relationship between pain and fatigue [28,31,32] in OA and other conditions, it is not surprising that fatigue is often considered a by-product of pain. However, when pain and fatigue are measured daily or within-day, differences emerge. For example, compared to samples of persons with RA and fibromyalgia, daily fatigue fluctuations were not as strongly associated with daily pain fluctuations in persons with OA [39]. In our work, we found that within-day fatigue was experienced at greater intensities than was pain [40]. Additionally, in one of the few studies to examine the symptom-activity relationship in OA, we found that fatigue was more highly associated with objective physical activity (PA; measured by accelerometer) compared to pain [40]. Lastly, we found differential effects for pain and fatigue by group when we tailored activity pacing [41], providing support for addressing pain and fatigue separately.

### Pacing Intervention Effectiveness

**Figure 1:**
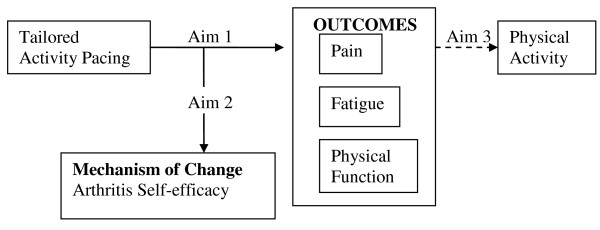
**Model of Symptoms, Self-Efficacy, Physical Function and PA in Adults with Osteoarthritis**.

Although activity pacing is a recommended non-pharmacological intervention for OA management based on consensus guidelines [4], its effectiveness as a stand-alone intervention is understudied. Typically, activity pacing is combined with other programs (e.g., coping skills training, cognitive behavioral therapy). In these studies of knee or hip OA, the overall programs have been effective at reducing pain [23,24]; however, it is impossible to disentangle the unique effects of activity pacing. Due to differences in how activity pacing is understood as a strategy, there is variability in activity pacing instruction and its delivery by therapists [42,43]. This trial addresses these limitations by using a standardized approach to teaching activity pacing to adults with knee or hip OA. This approach is structured around the overall goal of activity pacing, which is the balance between activity and rest in order to accomplish necessary and valued activities [44]. In this study, we will also be able to examine the unique effects of tailored activity pacing on fatigue for knee or hip OA in which our preliminary results showed significant improvements in fatigue compared to a general activity pacing intervention [41]. The conceptual model that guided intervention development is shown in Figure 1.

## Methods/Design

This is a three-arm randomized controlled trial that will examine outcomes at baseline, posttest (approximately 10 weeks post baseline), and 6 months. Ethical approval for this study was obtained by the University of Michigan Medical School Institutional Review Board and the Subcommittee on Human Studies in the Veteran's Affairs Ann Arbor Healthcare System. The overall goal of the study is to examine the effectiveness of a tailored activity pacing intervention for adults with symptomatic knee or hip OA. The primary outcomes are fatigue and pain.

The specific aims are:

Aim 1: To examine the effectiveness of a tailored activity pacing intervention on fatigue, pain, and physical function.

Aim 2: To determine if increased arthritis self-efficacy post intervention is related to improvements in symptom severity and function.

Aim 3: To evaluate the effect of tailored activity pacing on physical activity.

Eligible individuals are randomly stratified by age and gender into one of three arms: tailored activity pacing intervention, general activity pacing intervention, or usual care. The tailored and general activity pacing interventions are equivalent in session number and length. Participants are involved in study procedures over 6 months. For the intervention groups, involvement consists of 3 individual sessions, 3 testing visits, 3 home monitoring periods, 3 weekly follow-up phone calls, and monthly contact phone calls until the outcome assessment at 6 months. The usual care group participates in all activities described above except for the intervention sessions and the 3 weekly follow-up phone calls.

### Participant Recruitment

Community-living veteran and non-veteran participants are recruited from the University of Michigan and VA clinics, senior housing sites, and through public advertisements. Based on our previous studies, our optimal recruitment methods are ad placement at clinics and through community ads and fliers. As in our previous studies, areas with higher concentrations of minorities are particularly targeted for recruitment. Minorities such as African Americans are particularly important to include in this study because they not only have higher rates of knee and hip OA than Caucasians [45], they also report more severe OA symptoms and more physical disability even when controlling for demographics and other factors [46]. In addition, African Americans are less likely than Caucasians to opt for hip or knee replacements [47] and more likely to manage OA through home remedies that often have little to no evidence of effectiveness (such as copper bracelets or herbal medicines) [48].

### Eligibility Criteria

The following inclusion criteria were designed to identify a cohort of community-living adults who are likely experiencing symptoms specifically due to their OA. Prior to enrollment, each prospective participant is evaluated for inclusion. Table 1 lists the inclusion and exclusion criteria.

**Table 1:** Eligibility Criteria

Inclusion Criteria
• Age ± 50 years old • Pain for at least 3 months duration • Mild to moderate severity on the WOMAC pain scale • Radiographic evidence of knee or hip OA • Community-living (i.e, own home, senior residence, apartment) • Adequate cognitive status (score of > 5 on the 6-Item Screener)[66] • Ambulatory either with or without an assistive device • Reliable operation of the Actiwatch-Score accelerometer • English-speaking

**Exclusion Criteria**

• Medical conditions that may interfere with pain and fatigue reporting or activity monitoring (multiple sclerosis, lupus, rheumatoid arthritis, Parkinson's disease, peripheral neuropathy) • Cancer diagnosis in the last year (other than skin cancer) or currently undergoing treatment for cancer • Medicine changes within last 2 weeks • Anemia or unmanaged thyroid dysfunction (from blood work result) • 2 or more days of complete bed rest within last month • Limb hemiplegia or amputation • Knee arthroscopic procedure within the last 2 months • Replacement of any hip or knee within the last 6 months • Knee joint injection within the last 3 months • Current receipt of physical or occupational therapy for OA symptoms or knee/hip problems • Current attendance or attendance in the past 12 months in a cognitive behavioral program or other self-management program that includes activity pacing instruction.

### Procedures

**Figure 2:**
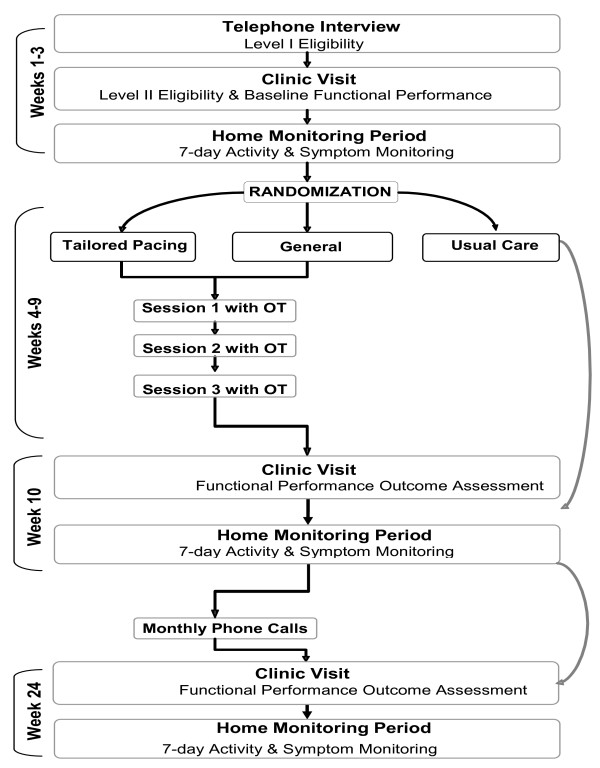
**Study Flow Chart**.

Figure 2 shows a flow chart of the study protocol. In brief, trained research personnel screen individuals for eligibility, administer performance-based assessments, and train participants in the use of the wrist-worn accelerometer and accompanying logbook. Participants provide written consent to participate in the study. They wear the accelerometer for a 7 day period and then return materials in person or by mail to the study team. Participants are randomly stratified by age and gender into the tailored activity pacing intervention, general activity pacing intervention, or usual care group. The usual care group participants undergo all outcome assessments and have maintained contact through monthly emails between outcome assessments similar to the intervention groups. The contact period between Weeks 11 - 23 is being undertaken to better ensure participation in the 6 month assessment for all groups. Each intervention arm involves 3 individual sessions with an OT. In the tailored activity pacing group, data collected from the home monitoring period are used to generate reports for participants about how symptoms relate to physical activity and are used by the occupational therapist. The general activity pacing group does not use the information from the home monitoring period during OT sessions.

### Study Arms

#### Usual Care Group

This group participates in all outcome measures and monthly outcomes are tracked during the study period using a survey measure to determine if any new OA pharmacological or non-pharmacological treatments are undertaken (e.g., injection, physical therapy, medication change) to treat symptoms.

### General and Tailored Activity Pacing Interventions

Regardless of whether people are assigned to the tailored activity pacing or general activity pacing intervention groups, participants meet with an OT for 3 individual sessions (total treatment time: 3 hours over Weeks 4 - 9 of the study). The first session for both the tailored activity pacing and general activity pacing includes a brief presentation by the OT on the nature of OA and how physical demand and mood can affect the symptom experience. This is followed by group-specific instruction on activity pacing as a tool for symptom management which includes printed materials that are a take-home reference for the participant. In the tailored activity pacing intervention, the OT discusses how to incorporate activity pacing based on the individual's profile of symptoms and activities determined from the baseline home monitoring period. In the general activity pacing intervention, the OT uses generic examples and does not mention the individual symptom experience from the baseline home monitoring period. By the end of the first OT session for both the tailored and general activity pacing groups, the participants develop a time-based activity plan with goals related to activity pacing. The second session focuses on individual progress with activity pacing, goal setting, and addressing barriers to using the recommended strategies. The third session focuses on reviewing activity pacing tenets, evaluating the use of time based activity pacing and planning for the future. After the 3 individual sessions have concluded, the OT makes 3 brief follow-up phone calls (1 call per week) to discuss activity pacing progress, symptoms, and any barriers.

### Strategies for Ensuring Treatment Fidelity

Treatment fidelity is defined as "the procedures used to monitor and enhance the reliability and validity of behavioral interventions [49]," and is an essential component of this study. Per recommendations from the National Institutes of Health (NIH) behavior change consortium [49,50], several strategies are being used to ensure treatment fidelity in this study. These strategies target fidelity of treatment delivery (standardized modules for the intervention and interventionist training and monitoring) and treatment receipt (i.e., participant involvement in treatment).

### Fidelity of Treatment Delivery

We have designed the study such that both intervention groups are equivalent in dose and administration of treatment (i.e., the number and length of sessions), including the manner in which participants are contacted from 10 weeks through 6 months. There are several procedures in place to ensure fidelity in how treatment is delivered. It is likely that there will be multiple therapists per active intervention arm (i.e., tailored activity pacing and general activity pacing). We decided that extra therapists per group could reduce the possibility of treatment effect due to therapist characteristics and ensure that we can deliver the intervention in the event that any interventionists drop out of the study. All therapists will undergo an initial training period which will involve structured education via a manual and a series of didactic sessions with the principal investigator (PI) and study team members. The initial training will also involve role playing in pairs and an observer who will rate the mock treatment session for key concepts to ensure consistency across therapists. Any discrepancies in how the intervention is delivered across therapists will be discussed and a consensus will be reached about intervention delivery.

To assess therapist adherence to the intervention protocol, they are asked to log the actual treatment time per session with each individual. The therapist also has a checklist of items for each visit to facilitate consistent presentation of the protocol. In addition, we are audiotaping sessions (as long as each individual consents to that process) and an independent rater listens to the tapes shortly after the sessions to determine if original criteria for intervention delivery in each treatment arm are being met. Any discrepancies in the audiotapes by therapists in a particular treatment arm will be resolved in a face-to-face meeting through consensus to reduce any variability in delivery. To prevent protocol 'drift', the PI and therapists in each arm have monthly telephone contact to discuss any issues and additional training sessions among therapists occurs as needed.

### Fidelity of Treatment Receipt

We will also assess participant factors related to treatment fidelity, i.e., how the treatment is received by the participant. This involves an assessment of individual factors such as comprehension of information, and the ability to use and perform skills associated with both the data collection and behavioral elements of the study. We have a standardized interactive learning module to teach the participant how to input symptoms on the wrist-worn accelerometer. Because the validity of the symptom information is central to our tailoring methods, all participants will undergo this learning module prior to their first session with the OT. In addition, both the tailored and general activity pacing interventions include strategies to ensure comprehension of the material. For example, participants are asked to reiterate main pacing concepts back to the therapist (e.g., importance of planning, resting before symptoms get worse, etc.). Participants also receive homework assignments that reinforce the lessons presented during the session and are asked to complete a daily activity logbook. Both of these homework items are used to assess personal compliance and overall protocol adherence. Each subsequent session involves a review of previously presented topics, a review of goals, and time for discussion of any barriers. Session 3 is focused on relapse-prevention, and continues the theme of setting goals and problem-solving to minimize barriers that impede the use of activity pacing. Lastly, we conduct debriefing interviews with each participant to examine their experiences in the intervention including adherence with activity pacing in daily life at the end of their involvement in the study.

### Measures

The primary outcome measures are fatigue and pain. Fatigue is assessed in two ways. First, fatigue is measured by the Brief Fatigue Inventory (BFI) [51], a reliable and valid measure of fatigue in adults with cancer. There is no gold standard measure of fatigue for adults with OA; however, the BFI is of particular relevance because it assesses fatigue by asking about interference in daily life, which is our primary interest. This scale is usually scored by calculating an overall score based on an average of 9 of the 10 items. In samples with rheumatic conditions, the BFI has recently been divided into two fatigue subscales (fatigue severity and fatigue interference) [52]. Although no studies have examined the clinical significance of these subscales in OA or in other samples, we are expecting similar or greater improvements in fatigue severity and fatigue interference as seen in our pilot study (8% and 15% improvement respectively) [41]. Second, because there is currently no commonly-used fatigue measure in OA across studies, an additional method of assessing fatigue from the Patient-Reported Outcomes Measurement (PROMIS) assessment tools will be used. PROMIS is a collection of carefully constructed item banks that can be used to assess multiple domains of relevance to complex and chronic illnesses. Due to the methods used to construct these item banks, such measures are expected to be more precise and less burdensome to patients to complete [53]. We are using the 8-item short form PROMIS fatigue scale [54]. Pain, the other primary outcome, is assessed by the Western Ontario and McMaster Universities Osteoarthritis Index (WOMAC). This is a validated, disease-specific questionnaire for adults with knee or hip OA in which participants rate their currently experienced pain and physical disability [55]. Based on previous studies, we will consider a 2 point decrease on the WOMAC pain scale to be clinically significant [56].

Other secondary outcomes and potential moderators include physical function, assessed by the 6 Minute Walk Test [57], a commonly used, validated measure of physical performance in adults; arthritis self-efficacy, the perceived ability to manage arthritis and pain and fatigue symptoms, measured by the Arthritis Self Efficacy Scale [58]; and other variables such as knee extension strength (as measured by a hand-held dynamometer), and general health status variables (e.g., BMI, self-reported chronic conditions, depression using the Geriatric Depression Scale [59], medication use). In addition, PA will be assessed by a wrist-worn accelerometer [Actiwatch-Score, Phillips Respironics-Mini Mitter Co, Bend OR] that measures changes in acceleration. Although it is worn on the wrist, it is highly associated with whole-body movement [60,61]. Changes in acceleration are recorded as activity counts and saved every 15 seconds. Higher activity counts reflect participation in higher intensity activities [62,63]. PA from the accelerometer will be aggregated in different ways. We will look at average activity counts and total activity counts that occur: 1) in the time between symptom reporting periods, 2) over each day, and 3) over the entire home monitoring period similar to our previous studies [40,41,64].

### Procedure for the Home Monitoring Periods

The accelerometer is mounted on each participant's non-dominant wrist. Participants are instructed on how to enter responses into the accelerometer using a standardized interactive learning module that we have developed and used in previous studies. Participants are given the opportunity to practice rating their symptoms and using the accelerometer's input button to record the information. If, for some reason, participants fail the learning module (e.g., inability to rate symptom experience on a numeric rating scale, inability to press the input button) they are excluded. Although we have not encountered exclusion for this reason in any of our past studies, this reporting is central to the tailoring portion of the intervention and therefore is an important criterion for study inclusion. Participants also become familiar with the logbook that accompanies the accelerometer and serves to cross-validate the items. The logbook is used as a back-up if there are missing data from the accelerometer.

Momentary pain and fatigue severity are measured on 0 -10 numerical rating scales that will be directly input into the accelerometer five times a day (at rise time in the morning, 3 times during waking hours, and at bedtime). The accelerometer is worn for 7 days at baseline and at the outcome assessment periods. Pain is rated on a scale from 0 = no pain to 10 = pain as bad as you can imagine. Fatigue, defined as tiredness or weariness [28], is rated on a scale from 0 = no fatigue to 10 = fatigue as bad as you can imagine. Momentary ratings are used in the study for the sole purpose of generating tailored reports on individual symptom-activity relationships during the baseline week for the tailored activity pacing intervention.

### Data Analysis

The target sample size in this study is 156 participants. In our recruitment plans, we have factored in a 30% attrition rate to achieve this number. Although there are three arms to the study: tailored activity pacing, general activity pacing, and usual care group, the study was powered to find effects between the two active intervention groups (tailored activity pacing and general activity pacing). Specifically, the power analysis was based on our pilot data in which we compared the tailored activity pacing intervention with the general activity pacing intervention and detected large group differences using the effect size Cohen's d [65] on BFI fatigue severity and interference (d = .79 and 1.1 respectively) at 10 week-follow-up. Based on a 2 group t-test at a significance level of p = .05, we will have greater than 90% power to detect an effect size of .74 or larger with 40 people in each of the two active intervention groups. Because most people with OA do not receive this type of intervention, it is important to compare the active intervention groups to a group receiving usual care. The study was powered to detect treatment effects between the active intervention groups; therefore we will also have sufficient power to detect differences between the active intervention groups and usual care group.

To examine the effectiveness of a tailored activity pacing intervention on fatigue, pain, and physical function, we will use repeated measures analysis of variance (RM ANOVA) models to analyze the responses (pain, fatigue, and 6 minute walk test) at baseline, 10 weeks, and 6 months for the three treatment groups (tailored activity pacing, general activity pacing, and usual care group). This will allow us to estimate the effects of treatment group, time point and the treatment × time interaction. Similarly, we will use RM ANOVA models to determine if increased arthritis self-efficacy post intervention is related to improvements in symptom severity and function and to evaluate the effect of tailored activity pacing on physical activity.

## Discussion

Although non-pharmacological treatment is recommended for people with knee and hip OA to manage symptoms, much of the evidence-base has examined these strategies in the context of a program of skills rather than individually. This study is examining the effect of a brief, individually-tailored, OT-led activity pacing intervention on pain and fatigue in adults with knee and hip OA. Given the positive effects of the tailored activity pacing approach compared to general activity pacing on fatigue in a small pilot study, we hypothesize that fatigue in particular will be improved with the tailored activity pacing intervention. We will also explore arthritis self-efficacy as a potential mechanism of change and the effects of pacing on PA at the 6 month follow-up period in an exploratory analysis.

## Competing interests

The authors declare that they have no competing interests.

## Authors' contributions

SLM, AKL, MEG, RLY, DJC, and DAW have made substantial contributions to conception and design of this study. SLM and AKL made significant contributions to acquisition of data, the data analysis plan, and interpretation of data. All authors have been involved in drafting the manuscript or revising it critically for important intellectual content. All authors have given final approval of the version to be published.

## Pre-publication history

The pre-publication history for this paper can be accessed here:

http://www.biomedcentral.com/1471-2474/12/177/prepub
